# Ndel1 Promotes Axon Regeneration via Intermediate Filaments

**DOI:** 10.1371/journal.pone.0002014

**Published:** 2008-04-23

**Authors:** Cory Toth, Su Yeon Shim, Jian Wang, Yulan Jiang, Gernot Neumayer, Camille Belzil, Wei-Qiao Liu, Jose Martinez, Douglas Zochodne, Minh Dang Nguyen

**Affiliations:** 1 Department of Clinical Neurosciences, University of Calgary, Hotchkiss Brain Institute, Calgary, Canada; 2 Department of Cell Biology and Anatomy, University of Calgary, Hotchkiss Brain Institute, Calgary, Canada; 3 Department of Biochemistry and Molecular Biology, University of Calgary, Hotchkiss Brain Institute, Calgary, Canada; Medical College of Georgia, United States of America

## Abstract

Failure of axons to regenerate following acute or chronic neuronal injury is attributed to both the inhibitory glial environment and deficient intrinsic ability to re-grow. However, the underlying mechanisms of the latter remain unclear. In this study, we have investigated the role of the mammalian homologue of *aspergillus nidulans* NudE, Ndel1, emergently viewed as an integrator of the cytoskeleton, in axon regeneration. Ndel1 was synthesized *de novo* and upregulated in crushed and transected sciatic nerve axons, and, upon injury, was strongly associated with neuronal form of the intermediate filament (IF) Vimentin while dissociating from the mature neuronal IF (Neurofilament) light chain NF-L. Consistent with a role for Ndel1 in the conditioning lesion-induced neurite outgrowth of Dorsal Root Ganglion (DRG) neurons, the long lasting *in vivo* formation of the neuronal Ndel1/Vimentin complex was associated with robust axon regeneration. Furthermore, local silencing of Ndel1 in transected axons by siRNA severely reduced the extent of regeneration *in vivo*. Thus, Ndel1 promotes axonal regeneration; activating this endogenous repair mechanism may enhance neuroregeneration during acute and chronic axonal degeneration.

## Introduction

Axonal injury, the underlying cause of spinal cord disability, neuropathies and neurodegenerative disorders, triggers changes in gene expression and protein complexes within neurons and glia. While some of these changes favor axonal regeneration, other signals drastically dampen the protective response, resulting in either failed or incomplete functional recovery. For instance, myelin-associated proteins and chondroitin sulfate proteoglycans impede axonal re-growth while neurotrophin retrograde signaling elicits neuronal survival [1]–[5].

Dynamic changes in axon structure constitute another critical path for nerve regeneration that can be triggered by modulation of the cytoskeleton, a highly organized architectural network composed of microtubules (MTs), intermediate filaments (IFs), microfilament (MFs) and their associated proteins [6]–[10]. Following neuronal injury, the developmental pattern of IFs resumes: IF proteins that decline during development are re-expressed [11]–[17]. This change in gene expression is thought to contribute to neuronal plasticity during regeneration. Nevertheless, the mechanisms of cytoskeletal remodeling within injured axons remain poorly defined.

Ndel1, the mammalian homologue of the *Aspergillus nidulans* NudE, is emergently viewed as an integrator and stabilizer of the cytoskeleton. In migrating neurons of the developing cortex, Ndel1 regulates MT dynamics and centrosome-nucleus coupling [18]. In mature CNS neurons, Ndel1 regulates Neurofilaments (NFs) assembly and homeostasis via a direct association with NF light chain (NF-L), thereby impacting neuronal survival [19]. Ndel1 also contributes to neurite outgrowth in PC-12 cells through interactions with the Disrupted-in-Schizophrenia protein1 (DISC-1) and Fez1 [20]–[22]. Recently, we found that Nde1l forms a molecular complex with the IF Vimentin and regulates Vimentin dynamics during neurite extension in CAD cells [23]. Vimentin also promotes neurite outgrowth in neuroblastoma and isolated primary neurons [24]–[27]. Consistently, Vimentin favors axon regeneration when present in neurons and Vimentin null mice exhibit impaired recovery of sensory response and reduced regeneration 6 days after sciatic nerve crush [27]). Nevertheless, Vimentin also displays anti-regenerative properties when expressed in glial cells [28]–[31]. Whether Ndel1 contributes to or impedes axon regeneration *in vivo* in association with Vimentin remains unknown.

We now discover that Ndel1 is upregulated at the mRNA and protein levels in injured axons *in vivo* and during regeneration, it preferentially associates with neuronal Vimentin. Remarkably, in a lesion induced neurite outgrowth assay of DRG neurons, and in rat models of sciatic nerve crush and transection, Ndel1 promotes axon regeneration. We propose that Ndel1 mediates regeneration via neuronal IFs.

## Results

### Axonal localization of the Ndel1/Vimentin complex

**Figure 1:**
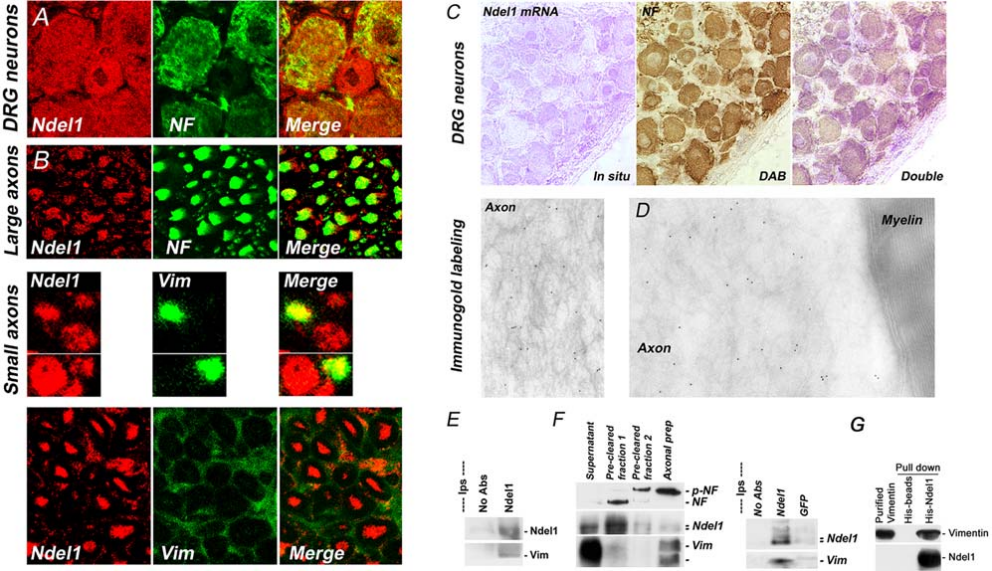
Axonal localization of Ndel1/Vimentin complex. (A) and (B) *In vivo* expression of Ndel1 in DRG neurons, large diameter NF-positive axons and small diameter Vimentin-positive axons but not in the Vimentin-positive basal lamina of glial cells. (C) Double in situ hybridization/immunohistochemistry depicting the expression of Ndel1 mRNA in NF-positive DRG neurons *in vivo*. (D) Immunogold labeling depicting the presence of Ndel1 on intermediate filament within the axon and its absence from myelin sheet. (E) Vimentin co-immunoprecipitates with Ndel1 in axoplasm. (F) Ndel1 and Vimentin co-fractionate in axonal cytoskeletal preparations. Vimentin co-immunoprecipitates with Ndel1 in axonal cytoskeletal preparation. The lower molecular form of Vimentin does not co-immunoprecitate with Ndel1 and may represent a hypophosphorylated form of the protein. All co-immunoprecipitations were performed using Ndel1 antibodies. No antibodies and GFP antibodies were used as negative controls. Pre-cleared fractions 1 and 2 contain non-axonal proteins (from cell body or other non-neuronal cells). (G) *In vitro* binding assay using purified His-Ndel1 and GST-Vimentin proteins. His-Ndel1 but not His-beads (negative control) pulls down GST-Vimentin.

Ndel1 and Vimentin contribute to neurite outgrowth, a read-out for axonal regeneration [20]–[22], [24]–[26]. Recently, we found that Ndel1 associates with Vimentin during neurite extension [23]. *In vivo*, Vimentin is expressed in both glia and neurons and therefore, the IF exhibits both pro- and anti-regenerative properties. To better understand the role of Ndel1 following axonal injury, we first determined the *in vivo* distribution of the Ndel1/Vimentin complex in nervous tissues (spinal cord, dorsal root ganglion (DRG) and sciatic nerve). As detected by confocal microscopy, Ndel1 protein was strongly enriched in DRG neurons and small and large NF-positive axons of spinal cord and sciatic nerve (Fig. 1A and 1B, 1^st^ row). Double *in situ* hybridization/immunohistochemistry with NF antibody further confirmed that Ndel1 mRNA was strongly expressed in DRG neurons (Fig. 1C). No *in situ* signal was found with the control sense probe (data not shown). Importantly, Ndel1 co-localized with Vimentin in a subset of small-diameter axons in these tissues but not with the Vimentin in the basal lamina of glial cells (Fig. 1B, 2^nd^ and 3^rd^ row). Consistent with the enriched localization of Ndel1 in neurons, Ndel1 antibodies conjugated to gold particles decorated the IF structures within sciatic nerve axon but were absent from myelin of Schwann cells (Fig. 1D).

**Figure 2:**
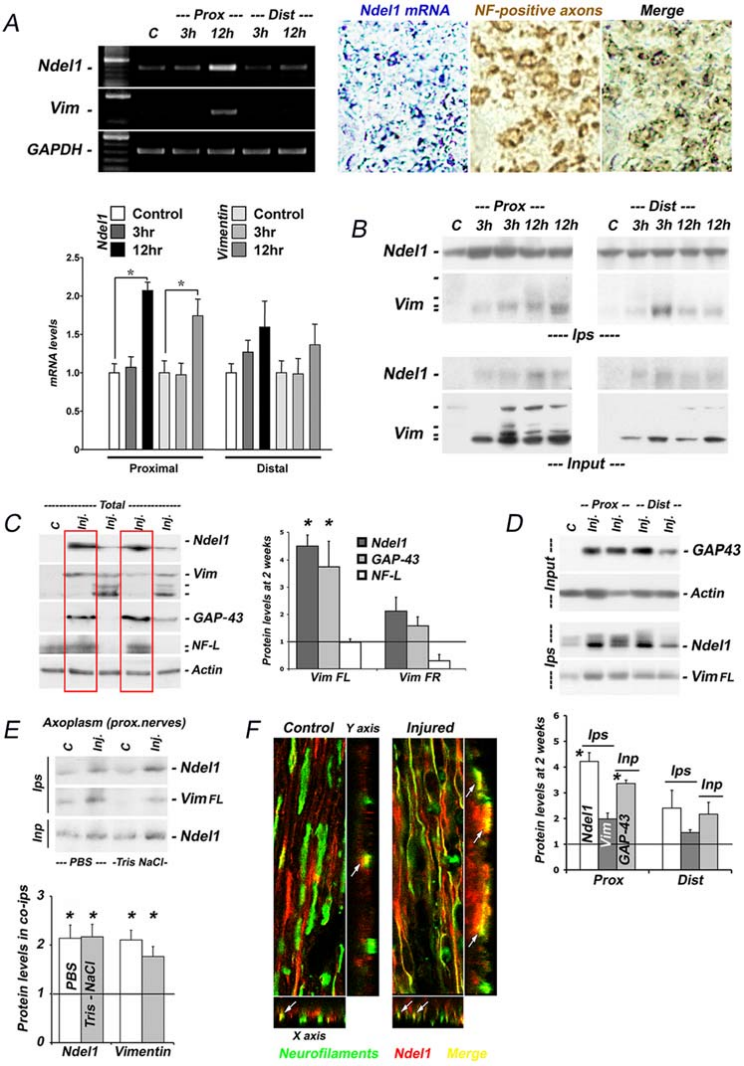
Upregulation and enhanced association of Ndel1 with Vimentin during axon regeneration in a sciatic nerve crush model. (A) Upregulation of Ndel1 and Vimentin in proximal stumps of crushed sciatic nerve 12 hours post-injury as revealed by RT-PCR. Quantification was performed twice on 3 samples for each condition (control, proximal -3h and 12h- and distal -3h and 12h-). GAPDH was used as control. * P (T<t) two tails: <0.001. Double *in situ* hybridization/immunohistochemistry revealed the local synthesis of Ndel1 (dark blue) in injured NF-positive axons (brown) 12h post injury. (B) Truncated fragment of Vimentin but not the full-length protein co-immunoprecipitates with Ndel1 early after injury (3–12h) in proximal and distal segments of crushed sciatic nerve (n = 5 for each condition). Note the early slight upregulation of Ndel1 protein versus the strong up-regulation and degradation of Vimentin full length. See supplementary figure 2 for quantifications. (C) Efficient axonal regeneration 2 weeks post crush as detected by total levels of GAP-43, a regeneration marker, and the mature neuronal marker NF-L, in nerve samples with the highest levels of Ndel1 and presence of full length (but not truncated) Vimentin (n = 8 for nerves with full length Vimentin; n = 5 for nerves with truncated Vimentin). Note the lower levels of NF-L in samples with slower regeneration. (D) Enhanced association of Ndel1 with neuronal Vimentin full length two weeks post-injury in nerve samples with robust regeneration as indicated by total levels of GAP-43 (n = 5 for each condition). Co-immunoprecipitations were performed with Ndel1 antibodies. (E) Enhanced association between Ndel1 and Vimentin in axoplasm of crushed nerves isolated in two different buffers (n = 5 for each condition). (F) Z-stack confocal pictures depicting the stronger co-localization of Ndel1 with small Neurofilament-positive axons in injured proximal segments of sciatic nerve (one week post-injury) vs non-injured nerves (n = 4). All co-ips were perfomed with Ndel1 antibodies. All quantifications in B to E were standardized to levels of non-injured nerves ( = basal level = 1); for panels C-E: * P (T<t) two tails: <0.0001 (compared to basal level). For all experiments, “ n ” indicated the number of nerve sample from individual animal. Vim FL: Vimentin full length; Vim TR: Vimentin truncated fragments; Ips: Immunoprecipitations; Inp: input.

To seek further evidence for an *in vivo* association between Ndel1 and neuronal Vimentin in neurons, we isolated axoplasms and axonal preparations from sciatic nerve and spinal cord, respectively. We took advantage of the neuronal pattern of expression of Ndel1 and performed co-immunoprecipitations on the preparations with Ndel1 antibodies in detergent-free buffers (see Material and Methods). In these conditions, Vimentin nicely co-immunoprecipitated with Ndel1 from PBS-isolated axoplasm of sciatic nerves (Fig. 1E). Furthermore, Vimentin also co-purified with Ndel1 in axonal cytoskeletal preparations from spinal cord and co-immunoprecipitated with Ndel1 in these preparations (Fig. 1F). The direct interaction between Ndel1 and Vimentin was demonstrated by an *in vitro* binding assay using purified His-Ndel1 and GST-Vimentin proteins. While His-beads did not pull down GST-Vimentin, His-Ndel1 pulled down GST-Vimentin (Fig. 1G). Taken together, these results indicate that Ndel1 and Vimentin selectively co-associate in a subset of axons *in vivo.* Of note, the Vimentin antibody detected a lower molecular band that may correspond to a hypophosphorylated form of the protein. It is also noteworthy that Peripherin, another IF expressed in neurons and involved in axon outgrowth and regeneration [13], did not co-immunoprecipitate and did not co-fractionate with Ndel1 in significant amount in spinal cord and nerves (Fig. S1). Based on the selective presence of Ndel1/Vimentin complex in axons and neurons (Fig. 2), we next determined whether the formation of the complex is enhanced following sciatic nerve crush and transection.

### Upregulation of neuronal Ndel1/Vimentin complex correlates with regeneration after sciatic nerve crush

Following sciatic nerve crush or transection, Vimentin is upregulated in both glial cells and injured axons/neurons at the mRNA and protein levels [12], [14]–[17], [27]. Using RT-PCR and Western blots, we first confirmed the upregulation of Vimentin within hours following sciatic nerve crush in rats. From 3 and 12 hours after injury, Ndel1 and Vimentin mRNAs and soluble proteins were synthesized and up-regulated in both distal and proximal stumps of injured nerves, when compared to the contralateral non-injured sides (Fig. 2). While Vimentin full length (FL) and reported truncated fragments were strongly upregulated, the augmentation in levels of Ndel1 protein was modest (Fig. 2A and 2B; Fig. S2 for quantification). The difference in upregulation of Ndel1 and Vimentin mRNA vs protein levels suggests that translation of these proteins is also regulated following sciatic nerve injury. As revealed by double in situ hybridization/immunohistochemistry, Ndel1 mRNAs were mostly found in NF-positive axons (Fig. 2A), consistent with its neuronal pattern of expression (Fig. 1). This is in contrast to Vimentin mRNAs that originate from both injured axons and glial cells [12], [14]–[17], [27].

To determine whether the Ndel1/Vimentin complex forms in injured axons, we again took advantage of the neuronal expression pattern of Ndel1 and performed co-immunoprecipitations with Ndel1 antibodies on sciatic nerve soluble lysates. We found that truncated forms of Vimentin, but not the full length, co-immunoprecipitated with Ndel1 at these early time points (Fig. 2B; Fig. S2 for quantification). These truncated forms have been suggested to participate in the early regenerative response (see discussion and [5], [27]).

Remarkably, the upregulation of Ndel1 and full length Vimentin was strongly sustained in injured nerves even up to two weeks after injury (Fig. 2C). The degree of augmentation in Ndel1 levels also correlated with higher levels of GAP-43, a neuronal marker for axonal regeneration, and with stabilization of NF-L, a marker for advanced regeneration and maturation (Fig. 2C). The stabilization of NF-L and increased GAP-43 did not correlate with the formation of the truncated fragments of Vimentin (Fig. 2C, graph). Importantly, in both proximal and distal segment of injured nerves, enhanced formation of neuronal Ndel1/Vimentin complex was observed after injury, as indicated by immunoprecipitations with Ndel1 antibodies (Fig. 2D). Highest levels of Ndel1/Vimentin in these co-immunoprecipitates were found in nerve samples with the highest degree of regeneration, as determined with levels of GAP-43 in total lysates (Fig. 2D). To further confirm the enhanced formation of this complex in axons, we isolated axoplasms from injured and non-injured proximal nerves and performed co-immunoprecipitations in detergent-free buffers (PBS or Tris-NaCl) using Ndel1 antibodies (see Material and Method). We found enhanced formation of the Ndel1/Vimentin complex in proximal segments of crushed nerves (Fig. 2E). The up-regulation of Ndel1 in regenerating nerve axons was further verified by co-staining with Neurofilament antibodies, followed by Z-stack sectioning on confocal microscopy (Fig. 2F). Morphologically, the regenerating axons often exhibited small diameter with higher Ndel1 content. Together, these results indicated that formation of the neuronal Ndel1/Vimentin complex is associated with axonal nerve regeneration.

### Upregulation of neuronal Ndel1/Vimentin complex correlates with regeneration after sciatic nerve transection

**Figure 3:**
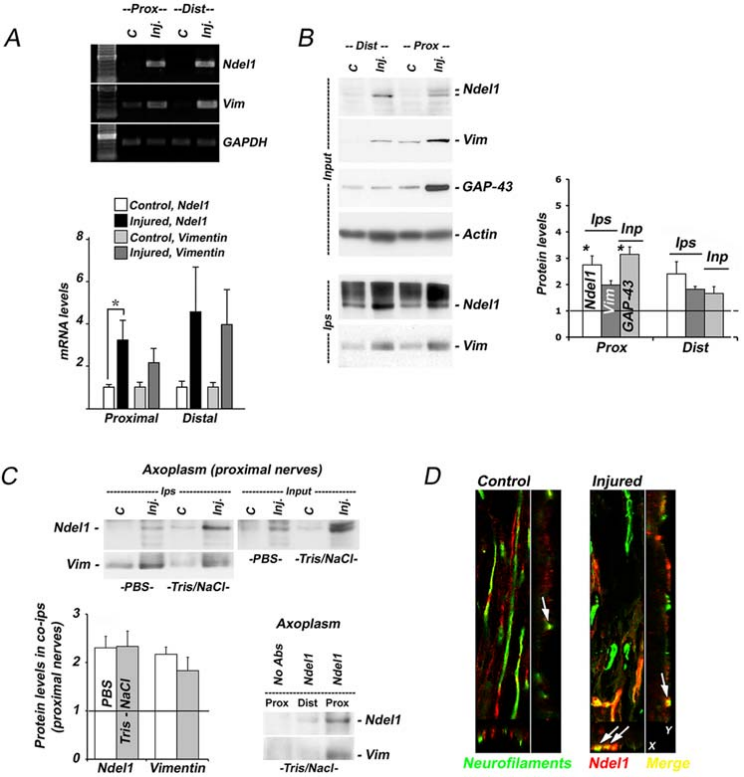
Upregulation and enhanced association of Ndel1 with Vimentin during axon regeneration in a sciatic nerve transection model. (A) Early upregulation of Ndel1 and Vimentin mRNAs in proximal and distal segments of transected sciatic nerve, 65 hours post-injury, as revealed by RT-PCR. Quantification was performed twice on 3 samples for each condition (control, proximal, injured, non-injured). GAPDH was used as control. * P (T<t) two tails: <0.05. (B) Enhanced formation of the neuronal Ndel1/Vimentin complex correlated with upregulation of GAP-43 in the proximal but not distal segment of transected nerve, 65 hours post-injury. Basal value = 1 for non-transected nerves. N = 5 for each condition. (C) Enhanced formation of the Ndel1/Vimentin complex in axoplasm of proximal segment of transected nerves as determined by co-immunoprecipitations with Ndel1 abs in two detergent-free buffers (PBS and Tris-NaCl). N = 5 for each condition. Note the stronger association of Ndel1 to Vimentin in axoplasm of proximal segment vs distal segment. Bar graph quantification is for the proximal segment. (D) Z-stack confocal pictures depicting the light and robust co-localization of Ndel1 with Neurofilament-positive axons in non-injured and transected proximal segments of sciatic nerve one week post-injury, respectively. All co-ips were perfomed with Ndel1 antibodies. Quantifications in panels B and C were standardized to levels of non-injured nerves ( = basal level = 1); * P (T<t) two tails: <0.0001 (compared to basal level). “ n “ represents the number of nerve sample from individual animals. Inp: Input; Ips: co-mmunoprecipitations, c: control; Inj: injured; Prox: proximal; Dist: Distal.

Next, we extended our findings to the rat sciatic nerve transection model. Similar to the crush model, we observed synthesis and upregulation of Ndel1 and full length Vimentin in both injured distal and proximal nerve stumps when compared to the non-injured contralateral sides (control) at 65 hours following sciatic nerve transection (Fig. 3A and B). The upregulation of soluble Ndel1 in injured nerves again led to enhanced formation of the neuronal Ndel1/full length Vimentin complex, as determined by co-immunoprecipitations with Ndel1 antibodies (Fig. 3B). The formation of the complex was corroborated with increased levels of GAP-43 specifically in the proximal fragment of the injured nerve still connected to nerve cell bodies (Fig. 3B). To further confirm the existence of this complex in axons, we isolated axoplasm from injured and non-injured nerves (distal and proximal) and performed co-immunoprecipitations in detergent-free buffers (PBS or Tris-NaCl) using Ndel1 antibodies. Again, we found enhanced formation of the Ndel1/Vimentin complex in injured axons, especially in the proximal segment (Fig. 3C). Using Z-stack sections of confocal microscopy, we further confirmed the upregulation of Ndel1 in injured regenerating axons (Fig. 3D and Fig. S2). Thus, the results obtained with both sciatic nerve crush and transection models (Figs. 2 and 3) strongly indicated that formation of the neuronal Ndel1/Vimentin complex is linked to axonal regeneration.

### Ndel1 associates differentially with IFs following injury

During axon regeneration, the IF expression pattern exhibited during neuronal differentiation resumes: Vimentin is re-upregulated while the NF Light chain (NF-L), another Ndel1-interacting protein in healthy axons, is downregulated [11]–[15], [17], [27], [32]. This change in IF expression is believed to favor plasticity of regenerating axons. Based on this knowledge, we reasoned that upon axotomy, Ndel1 will interact less with NF-L (in favor of Vimentin) in tissues with higher regenerative potential.

**Figure 4:**
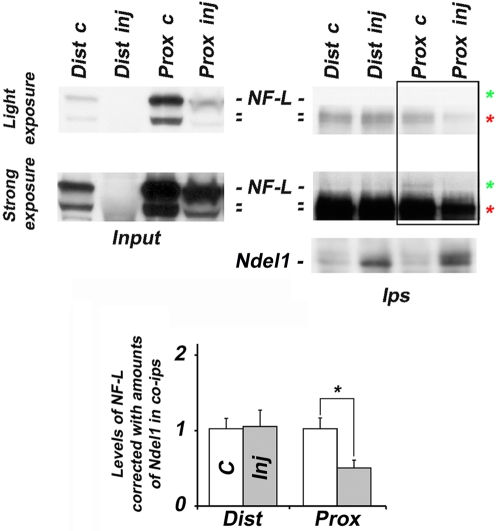
Ndel1 interacts differentially with NF-L in tissues with regenerative capability. NF-L isoforms interact less with Ndel1 in the proximal (but not distal) segment, 65 hours post-injury. Note that NF-L is downregulated in both segments after injury. Immunoprecipitations were performed with Ndel1 antibodies. Experiment was repeated 3 times on 4 different sets of distant and proximal segments. All co-ips were perfomed with Ndel1 antibodies. Quantifications were standardized to levels of non-injured nerves ( = basal level = 1); * P (T<t) two tails: <0.001 (compared to basal level). Ips: co-mmunoprecipitations, c: control; Inj: injured; Prox: proximal; Dist: Distal.

We first confirmed the downregulation of NF-L proteins (62 to 68 kDa) in both distal and proximal segments of injured nerves during the early stages of nerve axotomy (Fig. 4). Importantly, in the proximal segment of injured nerves where enhanced neuronal Ndel1/Vimentin association correlated with regeneration, there was reduced association between soluble Ndel1 and NF-L proteins when compared to the non-injured contralateral side (Fig. 4). In contrast, in the distal segment where little regeneration occurs, Ndel1 still associates with soluble NF-L proteins (Fig. 4). Together, these results indicate that following injury, Ndel1 interacts differentialy with soluble neuronal Vimentin and NF-L in nervous tissues undergoing regeneration.

### Silencing Ndel1 by RNAi in lesion conditioned DRG neurons reduces neurite outgrowth

**Figure 5:**
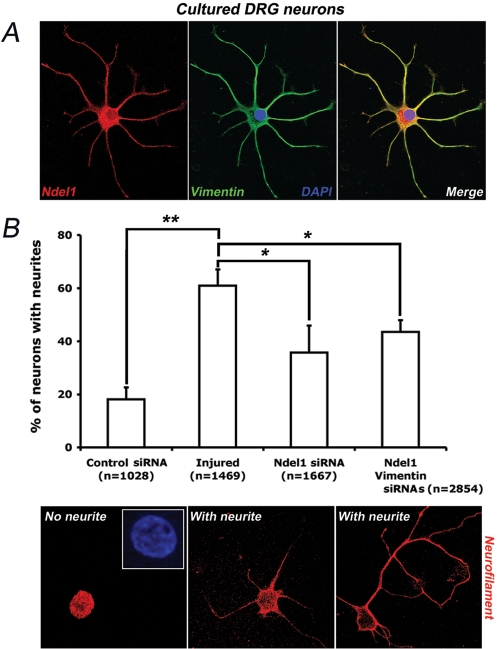
Silencing Ndel1 by siRNA reduces lesion-conditioned neurite outgrowth in DRG neurons. (A) Co-localization of Ndel1 and Vimentin in the cell body and neurites of nearly all Vimentin-expressing DRG neurons. (B) Reduction of the conditioning lesion-induced neurite outgrowth of isolated DRG neurons treated with Ndel1 siRNA. Co-treatment with Ndel1 and Vimentin siRNAs does not further impede outgrowth. 5 to 7 different DRG cultures were performed and the total number of neurons analyzed is indicated. Typical examples of NF-positive DRG neuron with or without neurites are depicted. Note that only DRG neurons with a normal nuclear morphology were counted. * P (T<t) two tails: <0.001, ** P (T<t) two tails: <0.0001. Red: NF; blue: DAPI.

To examine the role of Ndel1 in axon outgrowth following injury, we first investigated the conditioning lesion response of isolated DRG neurons treated with an Alexa 488-conjugated Ndel1 or control siRNA. We previously generated a specific siRNA against Ndel1 to knockdown the protein in primary neurons and mouse embryonic brains [18], [19]. An Alexa 488-conjugated scrambled sequence of siRNA without homology to any known protein was used as control. Sciatic nerve transections were performed 7 days prior to culture of neurons from L4/L5/L6 DRGs. Prelesioned conditioned neurons treated with Alexa 488-conjugated Ndel1 siRNA for 24 hours in culture incorporated the oligonucleotides (data not shown). Approximately 60% of cultured DRG neurons expressed Vimentin while nearly all Vimentin-positive DRG neurons express Ndel1. In these neurons, the two proteins co-localize in the cell body and neurites (Fig. 5A). Importantly, treatment of lesion-induced DRG neurons with Ndel1 siRNA reduced the number of neurons undergoing neurite outgrowth up to 50% as determined by staining with NF antibodies (Fig. 5B). Furthermore, co-treatment of lesion-induced DRG neurons with Ndel1 and Vimentin siRNAs did not further reduce the number of neurons extending neurites (Fig. 5B). These results indicated that Ndel1 is important for lesion-induced neurite outgrowth of DRG neurons *in vitro*. Based on data indicating that the two proteins associate together during neurite outgrowth [23], these results suggested that Ndel1 works upstream or at least serially with neuronal Vimentin in the same pathway.

### Local silencing of Ndel1 by siRNA in injured nerves reduces axon regeneration

**Figure 6:**
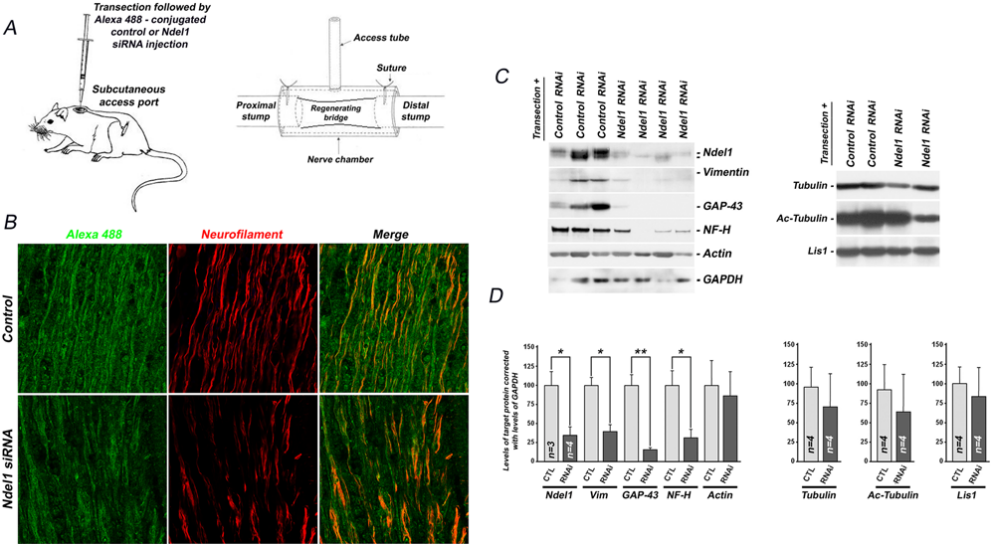
Silencing locally Ndel1 in transected axons by siRNA decreases regeneration. (A) Injection of the Alexa 488-conjugated control or Ndel1 siRNA usint the “T chamber” that connects the distal stump to the proximal stump. (B) Confocal picture showing the integration Alexa-488 control or Ndel1 siRNA in NF-positive axons of injected nerves. Note the reduced number of NF-positive fibers and dimmer Alexa 488 signal in the Ndel1 siRNA-injected nerves, indicative of reduced surviving axons. (C) Silencing Ndel in proximal stumps of transected axons by local injection of siRNA reduced expression of markers of regeneration (Vimentin, GAP-43) and axonal maturation (NF-H). Levels of Tubulin, Acetylated (stable), Tubulin, Lis1 and actin remain similar. GAPDH was used as standard. (D) Graph quantification of the levels of Ndel1, Vimentin, GAP-43, NF-H, Tubulin, Ac(etylated) Tubulin and Lis1 normalized with GAPDH or actin in arbitrary units. * P (T<t) two tails: <0.05, ** P (T<t) two tails: <0.001. Animals with transected nerves and injected with Ndel1 siRNA = 4; animals with transected nerves and injected with control (scramble) siRNA = 3. Western blot analysis was performed 3 times for each animal and condition (control and Ndel1 RNAi).

To directly demonstrate a role for Ndel1 in *in vivo* axon regeneration, we sought to silence locally Ndel1 by siRNA following sciatic nerve transection. Using the “T” chamber with the proximal stump connected to the distal stump [33], [34], we delivered the Alexa 488-conjugated Ndel1 siRNA to sectioned nerve via the injection port beginning at 2 hours after injury, with repeated injections 1, 3 and 5 days post injury (Fig. 6A). The mice were analyzed one week post transection. The injection port system has the advantage of locally silencing Ndel1 in injured axons. This approach allows us to target selectively injured axons and circumvents the early embryonic lethality of Ndel1 null mice. As indicated by double labeling with a NF marker and confocal microscopy, both control and Ndel1 Alexa-conjugated siRNAs were incorporated in the proximal stump of nerve axons (Fig. 6B). Nerves unexposed to injections were negative for Alexa 488 detection (data not shown). Importantly, the Ndel1 siRNA reduced by 50% the levels of Ndel1 protein indicated by western blots (Fig. 6C and D). Moreover, the Ndel1 siRNA but not control siRNA impeded axon regeneration as demonstrated by the reduction in NF-positive fibers, consistent with the decreased Alexa 488 signal (Fig. 6B). A remarkable downregulation of the regeneration marker GAP-43 and Vimentin was also observed in transected nerves injected with the Ndel1 siRNA (Fig. 6C and D). Consistently, levels of Neurofilament H (NF-H), a late marker for axonal maturation, were severely reduced in the Ndel1 siRNA-injected nerves. Together, these results indicated that Ndel1 plays a critical role in axon regeneration *in vivo*.

Finally, because Vimentin is expressed in both neuronal and glial cells, the *in vivo* injection of the Vimentin siRNA would affect both cell types. Therefore, there will be limitations in the interpretation of results obtained with these injections as well as co-injections of Ndel1/Vimentin siRNAs.

## Discussion

### Ndel1 is a new player in axon regeneration

The important role of Nde1l in neurite outgrowth and its association with Vimentin, an IF with dual role in axon regeneration, raised the question whether Ndel1 promotes or impedes axon regeneration *in vivo*. Ndel1 knockout mice are embryonic lethal [35] and therefore, can not be used to address this question. Using the McDonald “T” chamber, we locally silenced Ndel1 by siRNA in transected axons; we observed decreased regeneration *in vivo* (Fig. 6) in a manner reminiscent of, but to a more severe extent than sensory axons lacking Vimentin [27]. This was demonstrated by a reduction in levels of Vimentin and GAP-43, a marker for axonal regeneration, as well as NF-H, a late marker for axonal maturation in Ndel1 siRNA treated nerves (Fig. 6). It is unlikely that Ndel1 siRNA altered the Ndel1/Vimentin complex in glial cells as Ndel1 mRNAs and protein were found enriched in axons and neurons (Fig. 1). Furthermore, using multiple biochemical and cellular methods, Ndel1 was found to associate with Vimentin in axons (Figs. 2 and 3). If Ndel1 siRNA reduced Vimentin functions in glial cells, one should expect to see enhanced regeneration as Vimentin has anti-regeneration properties when expressed in glial cells [28]–[30], [36]. However, decreased regeneration associated with lowered levels of GAP-43, Vimentin and NF-H was observed. Finally, silencing Ndel1 by siRNA in conditioned lesion-induced DRG neurons also reduced axon outgrowth (Fig. 5). These data support the ideas that Ndel1 is in complex with Vimentin in axons and neurons and the Ndel1 siRNA altered the functions of neuronal Vimentin. A “residual” Ndel1/Vimentin complex in non-neuronal cells, if any, would have little effect on axon regeneration.

### Mechanisms of actions of Ndel1 during axon regeneration

By virtue of its association with molecular motors and its modulation of MTs and IFs [18], [19], [21], [22], [37]–[41], Ndel1 may remodel the cytoskeleton during axon regeneration. In our experimental conditions of injury, IFs were the most affected. Silencing Ndel1 by siRNA in injured axons significantly reduced the levels of IFs but left significantly unchanged the levels of Tubulin, Acetylated (stable) Tubulin, actin and the actin/MT-associated proteins FAK and Lis1 (Fig. 6). Compensation by other MT-associated factors may occur following injury.

During axon regeneration, the developmental pattern of IFs resumes: Vimentin is re-upregulated and expression of mature IFs such as NF-L and NF-H decreases [11]–[15], [17], [27], [32]. Our *in vitro* data suggested that Ndel1 works upstream of or at least serially with neuronal Vimentin in the regenerative pathway as co-silencing of Ndel1 and Vimentin does not further impede outgrowth of lesion-conditioned DRG neurons when compared to neurons treated with Ndel1 siRNA (Fig. 5). In tissues with higher regenerative capacity, Ndel1 preferentially associates with the neuronal form of Vimentin in detriment of NF-L. As neuroregeneration is being completed and NFs are re-upregulated to replace Vimentin, the ability of Ndel1 to associate with both Vimentin and NFs may confer to this protein the unique capacity to substitute IFs. For instance, through association with Vimentin and NF-L, Ndel1 may favor the formation of NF/Vimentin heteropolymers in injured axons, thereby serving as an initial site for polymerization of Neurofilaments to rebuild the IF network and stabilize the growing axons.

Of note, a recent study indicates that truncated fragments of Vimentin form a complex with phosphorylated ERK, Importin and Dynein and can elicit regeneration signals by scaffolding these proteins [5], [27]. We found that early hours after injury, Ndel1 co-immunoprecipitates with these truncated fragments but ERK and importins were not found in the co-precipitates (data not shown). These data may suggest heterogeneity in the molecular composition of the Ndel1/Vimentin complex in cells. Importantly, in nerves samples with slower regeneration, these truncated fragments of Vimentin were more abundant than the full length Vimentin. Our interpretation is that these fragments may participate in signaling at early phase of regeneration (0–12 hours) while the full length Vimentin acts at later stages of remodeling (days).

### Ndel1 and IFs null mice

The absence of phenotype in Vimentin null mice seems to argue against a role for Ndel1/Vimentin in neurite outgrowth and axon regeneration [42]. However, compensation for loss of Vimentin may occur. Alternatively, the ubiquitous removal of Vimentin may conceal the specific roles of neuronal Vimentin. For instance, *in vivo*, Vimentin may promote neurite outgrowth in developing neurons but may play an antagonistic role in other cell types; following gene knockout, the two opposite functions are cancelled, resulting in absence of overt phenotype.

Actually, Vimentin null mice exhibit subtle impaired recovery of sensory response 6 days after sciatic nerve crush and reduced levels of GAP-43, [27]. Mice lacking NF-L, another binding partner for Ndel1 differentially regulated during axon regeneration, exhibit delayed axon regeneration following sciatic nerve crush [32]. The lack of overt phenotype in these knockout mice strikingly contrasts with the embryonic lethality of Ndel1 null mice [35]. The essential role of Ndel1 during development and regeneration would be consistent with its upstream regulation on both Vimentin and NFs.

### Conclusion

In summary, our study reveals that Ndel1 is important player in axonal regeneration *in vivo*. Modulation of this pathway by upstream signals derived from both neurons and glia holds great promise to promote nerve regeneration For instance, the Ndel1 gene is controlled by CREB, a transcription factor that regulates neuronal activity and is induced during nerve injury for neuroprotection [43], [44]. In addition, pharmacological strategies aiming to manipulate the dynamics of neuronal Vimentin via Ndel1 may also directly contribute to intrinsic axonal growth during nerve regeneration.

## Materials and Methods

### Biochemistry

Total protein extracts of mouse spinal cord, sciatic nerve (distal, proximal stumps) were obtained by homogenization in SDS-urea β-mercaptoethanol (0.5% SDS, 8M urea in 7.4 phosphate buffer), Triton X-100 (10mM Tris-HCl [pH 7.5], 150 mM NaCl, 1 mM EDTA [pH 8.0], and 1% Triton) or E1A lysis buffer (50 mM Tris-HCl [pH 7.5], 250 mM NaCl, 5 mM EDTA [pH 8.0], and 0.1% Nonidet P-40) with a cocktail of protease inhibitors (PMSF, leupeptin, pepstatin, apoprotinin). For the soluble fraction, tissues were gently homogenized in the above buffers without detergent and the supernatant was collected after centrifugation at 10,000 g for 20 min or 15 min. Isolation of axoplasm was performed in detergent-free buffers (PBS or Tris-NaCl) by gently squeezing sciatic nerves and spinal cord as reported in[27]. The protein concentration was estimated by the Bradford procedure (Bio-Rad Laboratories, Hercules, CA). Immunoprecipitations were performed from isolated axoplasms in PBS or Tris-NaCl, cytoskeletal preparations and solubles fraction of spinal cord and sciatic nerves in the above described buffers without detergents (no SDS, Triton, Nonidet P-40). Proteins (20 to 50 µg) were fractionated on 7.5% SDS-PAGE and blotted on a nitrocellulose or PVDF membrane for western blot analysis. ) *In vitro* binding assay using purified His (1 µg), His-Ndel1 (1 µg) and GST-Vimentin (0,5 µg) proteins were performed according to [19]. Membranes were incubated with antibodies against Ndel1 (210, 211) [18], [19], Dynein intermediate chain (MAB 1618, Chemicon), actin (C4, MAB1501, Chemicon), Vimentin (Abcam, Chemicon), Peripherin (Chemicon), FAK (N-20, Santa Cruz Biotechnology), Acetylated-Tubulin (Abcam), Tyrosinated Tubulin (Abcam), GAP-43 (Abcam), Neurofilament light chain (Chemicon, Covance). The western blots were revealed by chemiluminescence (RENAISSANCE, Western blot kit, NEN Life Science (Boston, MA)).

### RNA isolation, RT-PCR and in situ hybridization

Total RNA samples from non-injured and injured sciatic nerve tissues (proximal and distal segments, with or without Ndel1 RNAi treatment) were isolated using the TRIzol Regent (Invitrogen) according to the manufacturer's instructions. The complementary DNA (cDNA) was reversely transcribed from same concentrations of total RNA products using random hexamers and M-MLV reverse-transcriptase (Promega). Quantitation of transcripts from the samples was performed by PCR with GAPDH primer. Each gene (Ndel1, Vimentin, GAP-43, and GAPDH) was amplified for 33cycles. 10 µl aliquots of PCR products were electrophoresed on a 1.5 % agarose gel in Tri-acetate-EDTA (TAE) buffer and visualized by staining with etidium bromide (EtBr). Primers for Ndel1, Vimentin, GAP-43, and GAPDH, are: Ndel1 up 5′ gcaggctgataaccaaagac 3′, Ndel1 dn 5′ ccgcagaatccatcttctc 3′, Vimentin up 5′ agatcgatgtggacgtttcc 3′, Vimentin dn 5′ tcttccatttcacgcatctg 3′, GAP-43 up 5′ ggctctgctactaccgatgc 3′, GAP-43 dn 5′ ctgtcgggcactttccttag 3′, GAPDH up 5′ gtgttcctacccccaatgtg 3′, GAPDH dn 5′ tgtgagggagatgctcagtg 3′. In situ hybridization using digoxigenin-labeled RNA probes was performed as described previously [45]. 16 µm DRG cryosections were fixed in 4% PFA and washed twice in PBT(PBS with 0.1% Tween-20). After bleaching with 6% H_2_O_2_/PBT and washing, sections were treated with 1 µg/ml proteinase K/PBT. Then sections were refixed and washed in PBT prior to hybridization overnight at 70°C. Sections were washed three times in 50% formamid/5xSSC/1%SDS at 70°C, followed by two washes in 50% formamid/2xSSC at 65°C. Staining was visualized using an NBT/BCIP (Roche) substrate.

### Generation, characterization of siRNA sequences and RNAi vectors

RNAi sequences were selected based on the criteria proposed by [46]. Complementary hair pin sequences or oligonucleotides were commercially synthesized and cloned into pSilencer 2.0 under promoter U6 (Ambion). Sequence for Ndel1 are base pair (bp) 276-294 (GCAGGTCTCAGTGTTAGAA) [18], [19]. A random sequence without homology to any known mRNA was used for control RNAi. All RNAi constructs were tested in 3T3 cells, CAD cells and primary neurons by both western blot and immunocytochemical staining. CAD cells were transfected with Lipofectamine 2000 (Invitrogen).

### DRG isolation and culture

L4/L5/L6 DRG neurons isolation was performed according to [47]. Briefly, DRGs were excised and roots and membranes were removed. After washing three times in cold L15, the DRGs were incubated for 1.5 hours at 37°C in L15 containing 1 mg of collagenase per ml. After pipetting the solution many times, the solution was centrifuged at 800 rpm for 5 minutes, followed by three washes in L15. Cells were resuspended in 1 ml of L15, and passed through a 70 micron mesh. Then the cell solution was loaded gently onto 2 ml of 15% BSA in L15 and centrifuged at 900 rpm for 10 minutes. The pellet cells were washed once in L15, and resuspended in advanced DMEM-F12 medium containing 1% N-2 supplement and 1% penicillin/streptomycin. Finally, cells were plated immediately on 6-well plate, previously coated with poly-L-lysine (0.1mg/ml). The plate was maintained in an incubator at 37°C with 5% CO_2_.

### Sciatic nerve transection and crush

65 Male Sprague-Dawley rats weighing 250-300g were used as an *in vivo* model for nerve regeneration. All procedures were approved by the Animal Care Committee of the University of Calgary in accordance with the Canadian Council on Animal Care. Sciatic nerve transection was performed at mid-thigh level on the left side under anesthesia with pentobarbital (65mg/kg, intraperitoneum injection). A T-chamber was placed in most of the rats undergoing this injury, as described below. Rats were given analgesic (butorphanol, 2mg/kg, subcutaneous injection) at 0.5–1 and four hours after the surgery to prevent autotomy and suture opening. Three days post surgery (72 hours), nerve tissues (proximal/distal stumps, 0.5–1 cm), the 5^th^ lumbar dorsal root ganglia (DRG), and the spinal cord dorsal column at the L5 spinal level) were harvested from both the transectioned and the untouched contralateral sides. Tissues to be used for mRNA detection were placed in Trizol , while tissues to be used for protein isolation and quantification were fresh frozen in liquid nitrogen. In both of these cases, tissues were maintained at −80 degrees Celsius post-harvest. Tissues to be used for immunohistochemistry, consisting of the proximal and distal segments of sciatic nerves were placed into Zamboni's fixative (2.0% PFA, 15% picric acid, 0.1 M sodium phosphate buffer, pH 7.3) or PBS PFA 4% overnight. The nerves were then washed three times in phosphate-buffered saline (PBS) and placed inside Cryomolds (Tissue-Tek). Then, the specimens were covered in Optimal Cutting Temperature compound (OCT, Tissue-Tek) and frozen at −80°C for at least one hour and kept frozen thereafter. In a second surgery, five rats had the left sciatic nerve crushed twice in succession, at angles perpendicular to one another, each for 30 s (total of 60 s) at a site 20 mm proximal to the trifurcation of the sciatic nerve branching, using a hemostat forceps. The site of injury was marked with a non-absorbent suture in adjacent muscle for later site identification. Animals were monitored post-operatively for signs of infection or other complications of surgery. Tissues were harvested as described above. Tissues were immediately processed for mRNA, protein isolation and immunoprecipitation. Tissues were processed as described above.

For later immunohistochemical investigations embedded nerves were cut into 14 µm sections using a cryostat (HM500, Microm) at −25°C and placed onto glass slides (SuperFrost White, VWR). On the day of staining, the tissues were post-fixed for 30 min at room temperature in 4 % paraformaldehyde in PBS, pH 7.4. After washing with PBS, the sections were incubated for 2 hr with blocking solution (PBS containing 3 % BSA and 0.2 % Triton X-100, pH 7.4). After rinsing, they were incubated overnight at 4 degrees Celsius in a humidified chamber with primary antibodies diluted in blocking solution. After washes with PBS, the sections were incubated for 2 hr with secondary antibodies (Jackson Immuno Research Laboratories) and 4′-6′-diamino-2′-phenylindole (DAPI, 2 µg/ml, Sigma) solution. The sections were mounted (Biomedia) and analyzed with confocal microscopy (Nikon).

### Delivery of siRNA in vitro and in vivo with the “T”chamber coupled to an injection port

The control (scrambled) siRNA or Ndel1 siRNA were commercially produced according to the manufacturer's protocol, with a fluorescent tag placed at the 3′ end with Alexa Fluor 488 (20 mM) (Qiagen, Canada). For DRG neurons, siRNAs were transfected for 24 to 48 hours using HiperFect Transfection Reagent (Qiagan, Mississauga,ON) according to the manufacturer's instructions. Shortly before transfection, injured DRG neurons were resuspended in Advanced DMEM (Invitrogen, Burlington, ON) and seeded on Poly-L-lysine coated coverslips in 6- well plates. siRNAs were diluted in 100 µl Advanced DMEM without serum and 16 µl of HiperFect transfection reagent was added to each of the diluted siRNA. The siRNA complexes were added dropwise on the cells and the plate was swirled gently to ensure even distribution of the transfection complexes. Immunofluorescence staining was performed according to Nguyen et al. 2004. For *in vivo* injections, 6.43 µl of siRNA (2000 ng or 120 nmol) were mixed with 20 µl HiPerfect Transfection Reagent and 73.57 µl Ringer's solution. An injection of 100 ul of siRNA preparations was administrated to each rat. The use of the “T” chamber coupled to an injection port has been described previously [33], [34]. The siRNAs were directly provided to the sectioned nerve and its surrounding milieu via the injection port first at 2 hours post-injury. Repeated injections were performed at 1, 3 and 5 days post-injury; with harvesting of the above tissues performed at 7 days post-injury.

## Supporting Information

Figure S1 No interaction between Ndel1 and Peripherin in spinal cord and nerves (A) Sucrose gradient demonstrating the significant co-fractionation of Ndel1 with Vimentin but not Peripherin in spinal cord and nerves. (B) Ndel1 does not co-immunoprecipitate with Peripherin and vice-versa in spinal cord and nerve lysates. HA antibodies were used as control for co-immunoprecipitations.
(2.67 MB TIF)

Figure S2 Protein levels 3 and 12 hours after sciatic nerve crush (A) Quantification graph of levels of Ndel1, Vimentin full length (FL) or truncated (TR) (in arbitrary units) 3 and 12 hours post injury in proximal (Prox) and distal (Dist) fragments (n = 5 for each condition). (B) Quantification of Vimentin truncated protein levels found in Ndel1 co-immunoprecipitates 3 and 12 hours post injury in proximal (Prox) and distal (Dist) fragments (n = 5 for each condition).
(5.19 MB TIF)
