# Supplementary figures and images for: Ndel1 Promotes Axon Regeneration via Intermediate Filaments
Source: PLoS One. 2008 Apr 23;3(4):e2014. doi: 10.1371/journal.pone.0002014 (PMC2291557; doi:10.1371/journal.pone.0002014)

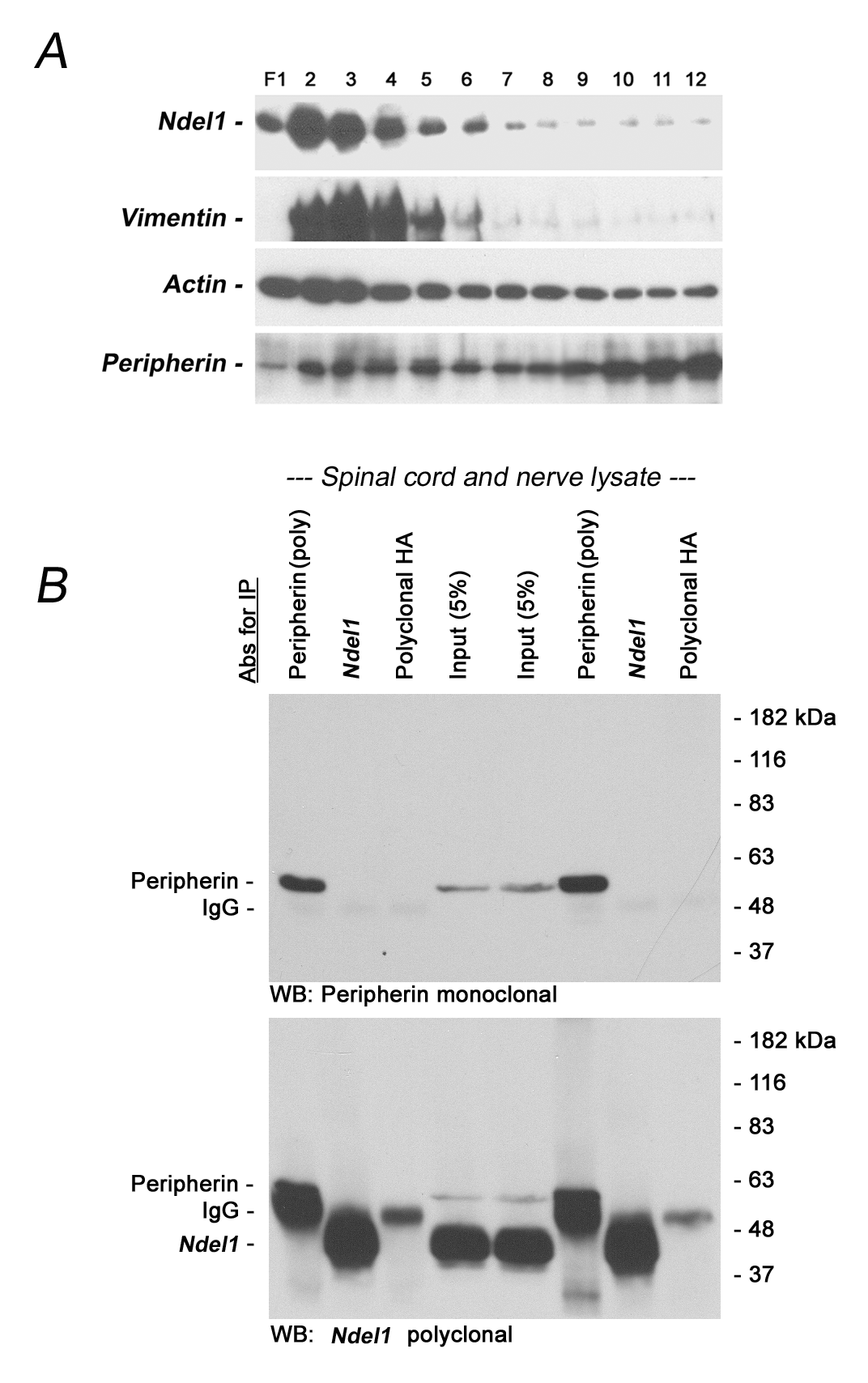

Supplement: Figure S1 — No interaction between Ndel1 and Peripherin in spinal cord and nerves (A) Sucrose gradient demonstrating the significant co-fractionation of Ndel1 with Vimentin but not Peripherin in spinal cord and nerves. (B) Ndel1 does not co-immunoprecipitate with Peripherin and vice-versa in spinal cord and nerve lysates. HA antibodies were used as control for co-immunoprecipitations. (2.67 MB TIF) [file pone.0002014.s001.tif]

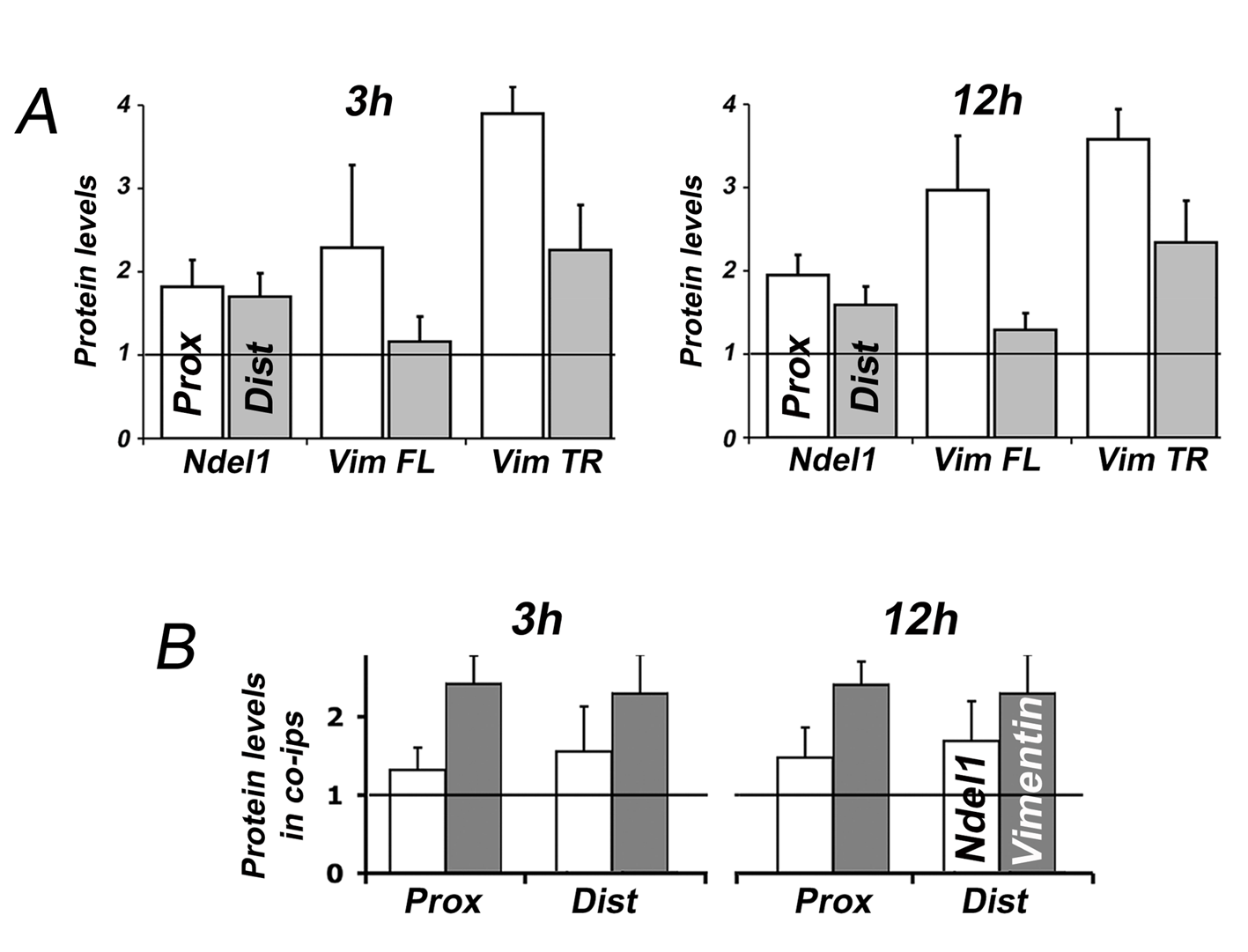

Supplement: Figure S2 — Protein levels 3 and 12 hours after sciatic nerve crush (A) Quantification graph of levels of Ndel1, Vimentin full length (FL) or truncated (TR) (in arbitrary units) 3 and 12 hours post injury in proximal (Prox) and distal (Dist) fragments (n = 5 for each condition). (B) Quantification of Vimentin truncated protein levels found in Ndel1 co-immunoprecipitates 3 and 12 hours post injury in proximal (Prox) and distal (Dist) fragments (n = 5 for each condition). (5.19 MB TIF) [file pone.0002014.s002.tif]
